# The Impact of Diet on miRNA Regulation and Its Implications for Health: A Systematic Review

**DOI:** 10.3390/nu16060770

**Published:** 2024-03-07

**Authors:** María DeLucas, Juana Sánchez, Andreu Palou, Francisca Serra

**Affiliations:** 1 Laboratory of Molecular Biology, Nutrition and Biotechnology (Nutrigenomics, Biomarkers and Risk Evaluation), University of the Balearic Islands, 07122 Palma, Spain; maria.delucas@uib.es (M.D.); andreu.palou@uib.es (A.P.); francisca.serra@uib.es (F.S.); 2 Instituto de Investigación Sanitaria Illes Balears, IdISBa, 07120 Palma, Spain; 3 CIBER Fisiopatología de la Obesidad y Nutrición (CIBEROBN), Instituto de Salud Carlos III (ISCIII), 28029 Madrid, Spain

**Keywords:** miRNAs, diet, dietary habits, metabolic diseases

## Abstract

The evidence suggests that diet can modulate endogenous microRNA (miRNA) expression. Changes in miRNA expression may affect metabolic processes and consequently be involved in health status and disease development. The aim of this systematic review was to summarize the evidence of the role of diet and specific food components in the regulation of miRNA expression and discuss its implications for human health and disease development. The PubMed, Embase and Web of Science databases were searched in accordance with the PRISMA (Preferred Reporting Items for Systematic Reviews and Meta-Analyses) guidelines for relevant studies. A total of 32 interventional and 5 observational studies performed in adults and evaluating dietary modulation of miRNA expression were included. Energy- and fat-controlled diets along with plant-based foods show substantial evidence of modulating endogenous miRNA levels. Plasma, serum and peripheral blood mononuclear cells (PBMCs) are the main sources used to measure miRNAs. A total of 108 miRNAs modulated by diet were identified. We confirmed that dietary habits are closely associated with the modulation of endogenous miRNAs. Particularly, energy content and fat intake appeared to be key factors influencing miRNA levels. Furthermore, since miRNAs are involved in the regulation of several biological processes, this modulatory process may affect health status and lead to metabolic disorders.

## 1. Introduction

Noncommunicable diseases (NCDs) such as obesity, type 2 diabetes and cardiovascular disorders have become a health problem of epidemic proportions. According to the World Health Organization (WHO), they are responsible for 71% of all deaths worldwide, corresponding to 41 million people per year, of which 15 million are premature deaths between the ages 30 and 69 [1]. Cardiovascular disease, cancer, respiratory diseases and diabetes are among the NCDs with the highest incidence [1]. Regarding the associated risk factors, along with tobacco, alcohol and physical inactivity, unhealthy dietary habits play a critical role, yet it is worth mentioning that all of these are preventable lifestyle aspects [2]. In recent decades, traditional diets have undergone a westernized shift toward overeating and the abuse of highly processed foods and added sugars, leading consequently to the exacerbation of NCDs [3]. On the other hand, NCD risk can be minimized or prevented by following healthy dietary habits, particularly when they are focused on normocaloric plant-based patterns [4], including a Mediterranean diet [5].

One of the reasons why diet influences the development of diseases is the participation of certain food components in the regulation of the metabolic processes involved [6,7]. Besides providing energy and nutrients, diet also contains bioactive compounds which can modulate biological processes, having an impact on health status. Recent data show that vitamins, polyphenols and fatty acids, as well as specific dietary patterns, regulate metabolism according to mechanisms involving the modulation of endogenous microRNAs (miRNAs) [8] and their expression [9,10,11]. Besides that, the evidence suggests that miRNAs contained in foods can also be absorbed during the digestive process and consequently interact with host gene expression [12,13]. A study assessing the presence of plant miRNAs in the serum of Chinese healthy adults, whose diet primarily consists of rice, has reported the detection of 30 exogenous miRNAs. Among these, significant levels of ath-miR-156a, ath-miR-166a and osa-miR-168a have been found, three miRNAs primarily derived from rice and cruciferous vegetables [12]. In another study that conducted bioinformatics analysis of data from four human small RNA libraries, 35 exogenous miRNAs have been detected in human milk exosomes, with the highest abundance levels observed for the plant-derived miRNAs ath-miR-166a, pab-miR-951, ptc-miR-472a and bdi-miR-168 [13]. Therefore, these studies suggest the potential capability of mature miRNAs to reach human plasma from the gastrointestinal tract and subsequently regulate the expression of human target genes. Due to the potential impact of miRNA modulation on human health and disease development [14,15,16,17], a full understanding of the interplay between diet, miRNAs and their health effects is needed.

miRNAs are endogenous small noncoding RNA sequences of approximately 22 nucleotides in length, which play a key role in the posttranscriptional regulation of gene expression [18]. During their biosynthesis, most miRNAs are first transcribed in the nucleus by RNA polymerase II (Pol II) into large pri-miRNA transcripts, which are cleaved into stem-loops of 70 nucleotides called pre-miRNAs by a complex formed by the RNase III enzyme DROSHA and the double-stranded RNA binding protein DiGeorge syndrome critical region gene 8 (DGCR8). Then, the pre-miRNAs are exported to the cytoplasm by Exportin 5 and cleaved into small double-stranded miRNAs 18–24 nucleotides long by the RNase III enzyme DICER, which is associated with TAR RNA binding protein (TRBP). These miRNA duplexes bind to argonaute proteins, assisted by ATP-dependent chaperone proteins. Subsequently, one of the strands is removed and degraded and the other one is loaded into the RNA-induced silencing complex (RISC), a ribonucleoprotein complex that intervenes in the recognition of the targeted mRNA [9,18]. Finally, this mature form of miRNA is guided to the 3′ UTR of the mRNAs through base pairing, leading to decreased mRNA stability and the repression of mRNA’s translation of target genes [19]. Potentially, each miRNA can modulate the expression of more than one target mRNA, and one mRNA can be modulated by several miRNAs, denoting an intricate miRNA–mRNA interaction network [20]. Additionally, miRNAs can be secreted out of cells and be stably transported into extracellular fluids associated with several carriers, including extracellular vesicles, ribonucleoproteins and lipoprotein complexes [21]. Consequently, miRNAs may have endocrine, paracrine and autocrine regulatory functions, contributing to cell-to-cell communication and participating in essential regulatory pathways involving apoptosis, differentiation, development, proliferation or signal transduction processes [19]. Therefore, disruption of the proper communication carried by miRNAs to cells has been related to the development of chronic disorders [21], including cardiovascular diseases [22,23], type 2 diabetes [24,25] and obesity [26,27].

In this context, animal studies have been successful in demonstrating the capacity of diet to regulate miRNA [28,29,30]. Although the evidence in humans is not as clear as in animal models, some studies have established a clear association between food intake and endogenous miRNAs. The aim of this systematic review is to summarize the evidence of the role of diet and specific food components in the modulation of miRNA expression and to discuss its implications for human health and disease development.

## 2. Materials and Methods

This systematic review was carried out following the recommendations of the Preferred Reporting Items for Systematic Reviews and Meta-Analyses (PRISMA) statement guidelines [31].

The PubMed, Embase and Web of Science (WOS) databases were manually searched on August 2023 to collect human studies which evaluated the impact of diet on miRNA expression. The search strategy used in PubMed and adapted to Embase and WOS was as follows: (diet OR dietary pattern* OR food OR intake OR dietary OR exogenous) AND (mirna* OR microrna*).

An initial selection of the returned articles was made by checking their suitability by evaluating their titles and keywords and then their abstracts. After this scrutiny, the full texts of the articles were analyzed to select those meeting the eligibility criteria (Table 1).

All the articles included in this review were summarized. The most relevant information was extracted and sorted according to the following items: (a) first author and publication date, (b) population of the study and (c) health status, (d) study design, (e) dietary strategy, (f) analytical method used to detect/quantify miRNA, (g) type of sample analyzed, (h) outcomes of the study.

Next, a network analysis was performed to characterize the biological functions of the miRNAs identified with further evidence on their association with diet. The miRNet 2.0 software [32] was used for the identification of target genes and, subsequently, for the determination of miRNA–function and miRNA–disease interactions. miRNAs with evidence in at least two studies were included in the network analysis, all of them with the prefix corresponding to the *Homo sapiens* (hsa-) species: hsa-mir-19b (MI0000074), -mir-19b-3p (MIMAT0000074), -mir-20a-5p (MIMAT0000075), -mir-21-5p (MIMAT0000076), -mir-29a-3p (MIMAT0000086), -mir-29b-3p (MIMAT0000100), -mir-92a (MI0000093), -mir-99a (MI0000101), -mir-99b (MI0000746), -mir-106a (MI0000113), -mir-106b (MI0000734), -mir-122 (MI0000442), -mir-122-5p (MIMAT0000421), -mir-130b (MI0000748), -mir-142-3p (MIMAT0000434), -mir-142-5p (MIMAT0000433), -mir-181a-5p (MIMAT0000256), -mir-181b-5p (MIMAT0000257), -mir-192 (MI0000234), -mir-192-5p (MIMAT0000222), -mir-221 (MI0000298), -mir-223 (MI0000300), -mir-328 (MI0000804), -mir-339-5p (MIMAT0000764), -mir-375 (MI0000783), -mir-411 (MI0003675), -mir-935 (MI0005757), -mir-1260b (MI0014197), -let-7b (MI0000063), -let-7c (MI0000064), -let-7f-5p (MIMAT0000067. The level of significance was set at *p* < 0.05.

## 3. Results

### 3.1. Characterization of the Included Studies

**Figure 1:**
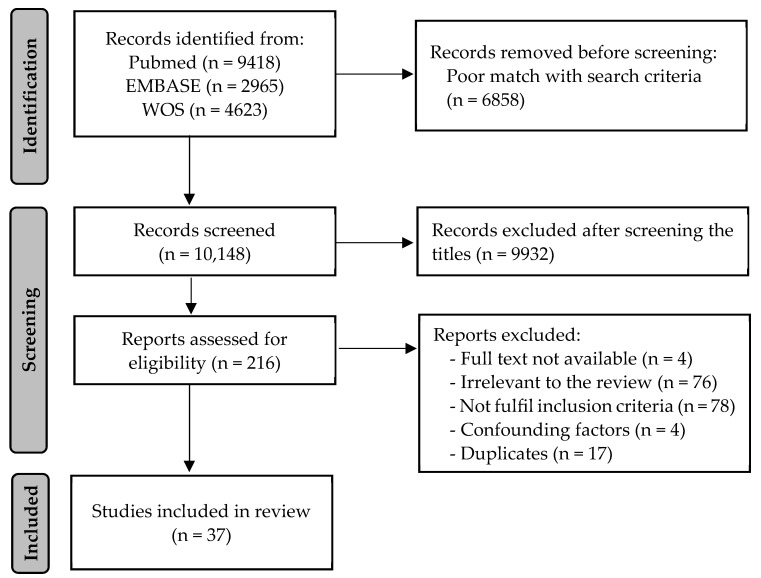
PRISMA flow diagram of the study selection process.

A total of 17,006 records were initially identified through the database search. The removal of records with a poor match with the search strategy turned out 10,148 entries. After the title and abstract screening, 216 manuscripts were considered suitable for eligibility. Finally, 37 articles met the inclusion criteria and were selected for this systematic review (Table 2). The detailed selection strategy is shown in Figure 1.

Among the 37 studies included in this review, 32 are interventional and 5 observational. Apart from that, 18 evaluate the association between certain dietary strategies and miRNA expression in healthy individuals, whereas the other 19 are focused on subjects with overweight or obesity. Most of the studies quantify the miRNAs in blood components (21 in plasma, 9 in serum and 4 in PBMCs [peripheral blood mononuclear cells]), but, beyond that, other specimens are used (3 studies analyze stool samples, 1 rectal mucosa, 1 subcutaneous adipose tissue, 1 sperm and 1 saliva). Quantitative PCR (qPCR) is the most common technique used to analyze miRNA expression; a total of 15 studies exclusively perform qPCR or a qPCR array, and 13 conduct a first screening followed by validation of the results using qPCR. The screening step is carried out using arrays (11 studies), RNA sequencing (2 studies) and performing qPCR with pooled samples (1 study). Additionally, five studies use only RNA sequencing, two the NanoString nCounter technology and one the miRNA sensor iLluminate.

Concerning the dietary strategies researched, the studies can be classified into four groups—energy-controlled diets [33,34,35,36,37,38,39,40,41,42,43,44,45,46,47], fat-related interventions [48,49,50,51,52,53,54,55,56,57,58,59], observational studies [60,61,62,63,64] and other dietary strategies [17,65,66,67,68]—which are further developed in the next sections. Articles examining the uptake of exogenous miRNAs from dietary sources were deemed insufficient for inclusion in the review. Nonetheless, given the emerging potential of food-derived miRNAs, their implications for health have been discussed below.

Regarding the miRNA nomenclature, some articles refer to the precursor miRNA and others to the mature form. Besides that, some authors do not use a letter after the number of the miRNA, which differentiates members of the same family, or the suffixes -3p and -5p to indicate from which double-stranded RNA the mature sequence comes. For these reasons, the miRNAs included in this review have been analyzed by family, grouping miRNAs with a similar structure and evolutionary origin [69]. Following this criterion, there is evidence that 108 miRNA families are modulated by diet. Despite that, only 37 of them report significant results in more than one study. The most relevant ones are shown in Table 3.

### 3.2. Modulation of miRNA Expression by Energy-Controlled Diets

A total of 15 studies evaluate the impact of energy-restricted diets on miRNA expression [33,34,35,36,37,38,39,40,41,42,43,44,45,46,47], of which 2 are energy-restricted diets without a weight loss purpose [33,34], 10 are weight loss interventions [35,36,37,38,39,40,41,42,43,44] and 3 consider fasting periods [45,46,47]. The characterization of the effects of energy-restricted diets on miRNA expression is mainly performed in healthy adults with overweight or obesity, although three studies also include subjects of a normal weight [35,37,45]. Regarding the experimental design, most of the studies include an intervention period of dietary restriction greater than 8 weeks, except for the three fasting studies [37,45,46], which lasted 5, 10 and 28 days, and two energy restriction studies of 4 and 6 weeks [34,39]. Although there were important differences between the diets implemented and their caloric content, they all agreed on caloric restriction, varying the range of restriction from a reduction of 30% or 500 kcal/day to periods of fasting for several days.

A total of 46 miRNAs have been linked to energy-restricted diets, although only 12 of them have been studied in more than one article (miR-19, miR-22, miR-29, miR-99, miR-122, miR-126, miR-142, miR-221, miR-223, miR-411, miR-935, let-7). In this respect, miR-19 is down-regulated in two fasting studies [46,47] and miR-99 in two energy restriction programs [33,39]. Additionally, miR-142 is down-regulated in responders to a low-fat diet [35] and after a 10-day fasting period [46]; the up-regulation of miR-126 is found after a fasting period [46] and also after a 12-week weight loss diet with a deficit of 500 kcal/day [38]. Interestingly, the subjects who lost less weight after an energy-restricted diet [41,43] were the ones who showed higher levels of miR-935.

Studies involving the remaining miRNAs showed a more complex pattern of regulation. miR-22 is up-regulated in responders (weight loss > 5%) to a 16-week intervention with a low-fat diet [35] but down-regulated after a 10-day fasting period with a daily intake of 250 kcal [46]. miR-122 is down-regulated in subjects who follow a 12-week weight-loss diet with a deficit of 500 kcal/day [38] and after a 12-month weight loss program [36] but also up-regulated in adults who have undergone a 10-week fasting intervention [46]. Concurrently, miR-411 and the let-7 family are down-regulated by energy-restricted diets [33,39] but up-regulated after a fasting period [45,47].

Concerning miR-29 and miR-221, both were down-regulated in the plasma of responders to a weight loss low-fat diet of 16 weeks [35], but other studies show up-regulation related to weight loss diets; miR-29 is up-regulated in the subcutaneous adipose tissue after a 15-week intervention [40], and miR-221 is induced in the plasma of both responders and non-responders after a 16-week intervention [43]. Different outcomes are reported on miR-223 after a weight loss diet depending on sex and the sample analyzed; this miRNA is up-regulated in the plasma after 16 weeks of intervention regardless of the sex of the population [43] but also down-regulated in the serum HDL fraction of men after 12 weeks of intervention [44]. Lower levels of this miRNA have also been seen at baseline in the PBMCs of women who do not respond to 8 weeks of dietary intervention [41].

### 3.3. Modulation of miRNA Expression by Fat Intake

Twelve studies analyze the impact of different fat-related diets [48,49,50,51,52,53,54,55,56,57,58,59]. Three of them consist of a single high-fat meal [48,49,50], five evaluate nut intake [51,52,53,54,55], two analyze extra virgin olive oil (EVOO) intake [56,57], one a ketogenic diet [58] and one the intake of trans fatty acids [59]. The last report is the only one with no significant results. These studies measure the miRNAs in blood components, except one article about miRNAs in sperm [55]. The method used to analyze miRNAs was qPCR, with an initial screening in some of them [48,51,52,53,55,57], except for one study, which used the NanoString nCounter technology [58].

Two interventional studies evaluate the impact of a single high-fat and high-energy meal on postprandial miRNA expression [48,50], but they analyze different miRNAs. One of them reports nine miRNAs down-regulated (miR-613, miR-629, miR-24-2, miR-555, miR-148a, miR-621, miR-875, miR-513c, miR-1226) and nine up-regulated (miR-653, miR-19b-1, miR-363, miR-885, miR-339, miR-938, miR-148b, miR-593, miR-200b) after a high-fat meal of 800 kcal [48], and the other one finds three miRNAs down-regulated (miR-1260a, miR-92b, miR-205) and six up-regulated (miR-200c, miR-143, miR-200b, miR-143, miR-375, miR-145) after a high-fat meal of 1067 kcal [50]. The miR-200 family is up-regulated after both single-meal studies. Another study using a similar dietary strategy but adding orange juice, a glucose drink or water in a crossover model observes the up-regulation of miR-375 after the high-fat meal with orange juice and down-regulation of miR-205 after the meal with the glucose drink [49].

Regarding the five studies analyzing nut consumption, their interventions lasted at least 8 weeks, and the study population was healthy adults of a normal weight in three of them [51,52,55] and with overweight/obesity in the remaining two [53,54]. Daily intake of 30–60 g of walnuts results in the overexpression of miR-32 [52], miR-29b [52] and miR-551a [51] when compared with the controls who abstained from walnuts [52]. Additionally, the down-regulation of miR-328, miR-330, miR-221 and miR-125a and the up-regulation of miR-192, miR-486, miR-19b, miR-106a, miR-130b, miR-18a and miR-769 are observed after 8 weeks with an almond and walnut intake of 30 g/day [53]. Furthermore, spermatic miR-34b is down-regulated after nut intake when comparing a Western diet avoiding nuts with a Western diet with 60 g/day of nuts [55].

Regarding EVOO intake, both studies evaluate the impact of a single dose of EVOO on the PBMCs [57] and plasma [56] and observe the modulation of several miRNAs. Interestingly, one of them reports the underexpression of miR-192 [57] and the other one overexpression [56]. Beyond that, the ketogenic diet study reports the underexpression of circulating miR-504 and the overexpression of let-7b and miR-143 after 6 weeks following this high-fat low-carbohydrate dietary pattern in a cohort of 12 healthy adults [58].

### 3.4. Dietary Patterns Related to miRNA Modulation in Observational Studies

Among the observational studies [60,61,62,63,64], four of them evaluate differences between vegan, vegetarian and omnivorous diets [60,61,62,63], and the other one is on the Mediterranean diet [64]. Regarding the differences between vegan, vegetarian and omnivorous diets, one of the studies does not report significant results [60]. The up-regulation of miR-92a in stool and plasma [62] and the down-regulation of miR-636, miR-4488 and 4739 in stool [63] are observed in association with vegan and vegetarian diets [62]. Additionally, in a cohort of 96 healthy adults, miR-3661, miR-320c, miR-29a, miR-320b and miR-204 are overexpressed and miR-132 underexpressed in the plasma of subjects who follow vegan or vegetarian diets [61]. Concerning the impact of the Mediterranean diet on miRNA expression, adherence to this dietary pattern is associated with higher levels of miR-590 in adults with obesity [64].

### 3.5. Other Dietary Patterns Related to miRNA Modulation

The five remaining studies evaluate the intake of high-red meat [17], protein consumption [65], a Korean diet [66], grape intake [67] and orange juice consumption [68].

A randomized crossover study design, with 23 healthy volunteers, studies the influence of two 4-week dietary interventions: a high red meat (HRM) diet and an HRM diet + supplementation with butyrylated resistant starch on miRNA expression in rectal mucosa. The up-regulation of miR-19a, miR-19b and miR-21 after the HRM diet and down-regulation of miR-17, miR-19a, miR-19b, miR-20a and miR-92a when adding resistant starch are reported [17]. Besides that, the association between protein consumption and circulating miRNA expression is analyzed in a group of three healthy men over the age of 70 [65]. After 2 weeks of study, a high protein intake (1.6 g/kg body weight/day) is associated with the underexpression of miR-125b, miR-100, miR-99a, miR-23b and miR-203 [65].

Apart from that, a 2-week intervention study evaluates the differences between a Korean diet and a westernized Korean diet in circulating and salivary miRNA expression in a group of 10 women with overweight and concludes that the Korean diet down-regulates miR-26a and miR-126 in the plasma and miR-92 and miR-122 in the saliva, while the westernized diet down-regulates miR-25 in the plasma and miR-31 in the saliva [66].

The effects of the intake of 5 g/kg body weight/day of fresh grapes on circulating miRNA expression are analyzed in a cohort of 40 adults with overweight; the up-regulation of two miRNAs (miR-208a and miR-33a) and down-regulation of 18 miRNAs (miR-181a, miR-30e, miR-30d, miR-335, miR-222, miR-15a, miR-421, miR-339, miR-378a, miR-29b, miR-106b, miR-324, miR-1260a, miR-365a, miR-155, miR-335, miR-200c and let-7f) have been observed [67]. let-7f, together with miR-126, is also down-regulated in the PBMCs after 4 weeks of consuming 500 mL of orange juice per day, while miR-144, miR-424 and miR-130b are up-regulated [68].

## 4. Discussion

Taking together the results of the included articles, the data confirm the involvement of several dietary patterns, as well as specific foods, in the modulation of miRNA expression in human cells, which is mainly reflected in plasma levels. However, the studies collected are quite heterogeneous. The interventions show large differences in terms of their duration and dietary strategy, as well as the characteristics of the selected study population, such as sex and age, with both parameters closely linked to the expression of several miRNAs [70]. The method used to quantify miRNA expression is also worth noting when interpreting the results, as each one provides different information. Three kinds of quantification methods have been identified: targeted analysis, sequencing and a combination of both. Targeted analysis using qPCR is the most used and offers high sensibility and specificity for detecting low levels of miRNAs [71] but has the drawback of quantifying a limited number of miRNAs, which are chosen by researchers following a hypothesis-driven approach. In contrast, miRNA sequencing provides extensive information about the entire miRNAome but has a lower sensibility in detecting those miRNAs expressed in small amounts [72]. Therefore, as some authors have opted for, the development of studies that use a combination of both methods might be advisable. This involves an initial screening using miRNA sequencing, followed by a targeted analysis of the selected miRNAs using qPCR to validate the observations. This approach would bring more reliable results showing not only how specific miRNAs are influenced by diet but also the entire miRNAome. Notwithstanding these limitations, the available data provide enough evidence to point out specific dietary patterns capable of modulating miRNA expression, which are discussed below.

### 4.1. Influence of Energy Intake on miRNA Regulation

Energy-controlled diets have been widely studied in the context of miRNA regulation, mainly with weight loss purposes. Regarding its consequences, we should differentiate the outputs obtained when considering the duration of the intervention from those based on feeding conditions.

**Figure 2:**
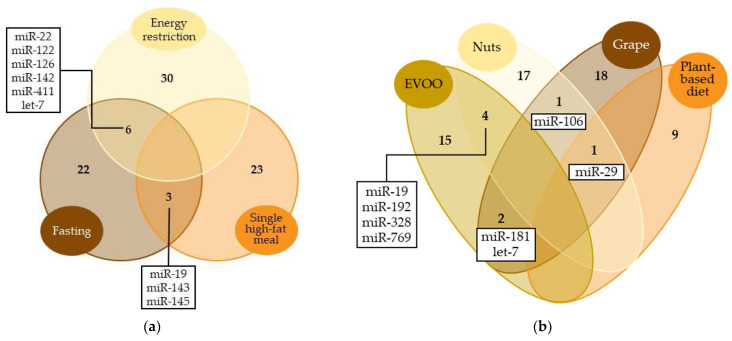
Venn diagram comparing the most representative miRNAs modulated by (**a**) energy-controlled dietary patterns and (**b**) plant-based foods. Only miRNAs with significant results in at least two studies were included. Numbers represent the count of miRNAs modulated by each dietary pattern. miRNAs modulated by ≥2 dietary patterns are specified.

Given the extensive study of hypocaloric diets in miRNA regulation, most of the miRNAs mentioned in this review were associated with energy restriction, either due to long-term daily energy restriction or shorter interventions (Figure 2a). For instance, the up-regulation of miR-126 [38,46] and down-regulation of miR-142 [35,46] have been related to both intervention models [38,46]. Occasionally, the effects observed on the miRNA levels vary depending on the duration of the restriction. This, together with the fact that the experimental designs tend to be quite different, makes it difficult to analyze the outcomes jointly. That is the case with let-7 [33,39,45], miR-22 [35,46], miR-122 [36,38,46] and miR-411 [33,47], whose evidence reported both up- and down-regulations associated with energy restriction. In these cases, some authors reported short-term consequences [46] and others the opposite, long-term outcomes [35,46]. The durations of the interventions prevented us from merging the results.

Regarding the feeding conditions, differences in miRNA levels have been observed between fasting and the postprandial state. The up-regulation of circulating miR-19, miR-143, miR-145, miR-200, miR-339 and miR-375 and the down-regulation of miR-92, miR-205 and miR-1260 have been seen in the postprandial state after a single high-energy and high-fat meal when compared with the fasting state [48,49,50]. In line with these results, another study has reported decreased levels of miR-19, miR-143 and miR-145 after a fasting period [46,47]. Apart from that, some of the changes in miRNA expression associated with hypocaloric diets might be influenced by macronutrient content. One example is miR-221, since it is down-regulated with a hypocaloric and low-fat diet [35] and up-regulated with a restricted diet which does not take into account fat content [43]. Fat intake may therefore be implicated in miR-221 modulation and, as the evidence associates miR-221 with insulin levels, it might also be involved in the development of insulin resistance [73,74]. This is the same for miR-223 outputs, as it has shown down-regulation in serum HDL fractions after a weight loss diet high in protein [44] and up-regulation in the plasma after a hypocaloric diet disregarding protein content [43]. The biological sample selected to measure the miRNA levels may also influence the differences between the aforementioned findings. Several miRNAs show tissue specificity, and large differences are found among the miRNA spectrum of several body fluids [75]. This may imply different tissue-dependent effects but with a common purpose [76]. In view of the above, the development of further studies with similar experimental characteristics may help strengthen the evidence available.

Interestingly, miRNA levels have been related to the response to diet-induced weight loss. Non-responders to a weight loss diet presented overexpression of miR-935 and miR-4772 and underexpression of miR-223, miR-224 and miR-376 before the dietary intervention [41,43]. These miRNAs may be considered biomarkers of weight loss susceptibility.

Considering the results of these studies, we could say that energy intake was a major regulatory factor in the human miRNA profile. Changes in the caloric content of the diet lead to the modification of endogenous miRNA levels, and the effects may vary depending on the duration of the intervention and the feeding conditions. Furthermore, the influence of these miRNAs over metabolic pathways and their implication in the development of metabolic diseases has been proposed. For instance, the evidence shows the involvement of miR-22 in the control of metabolic homeostasis [77]. Silencing of this miRNA has been suggested in the treatment of metabolic diseases, including obesity and hepatic steatosis [77,78]. Along with this, miR-122 participates in the hepatic metabolism of lipids, regulating the expression of genes involved in cholesterol and fatty acid synthesis [79]. Additionally, miR-19 and let-7 play a role in insulin signal transduction and have been related to the development of type 2 diabetes and obesity-induced insulin resistance [79]. miR-143 has also been associated with glucose and lipid metabolism [79,80]. These findings reinforce our knowledge about the involvement of energy intake-modulated miRNAs in the regulation of several biological processes [18,81] and their subsequent implications for the development of metabolic diseases [82,83,84,85].

### 4.2. Influence of a Mediterranean Diet and Plant-Based Foods on miRNA Regulation

The Mediterranean diet is widely known for its health benefits, as most of its characteristic food components have been attributed anti-atherosclerotic, anti-inflammatory or antioxidant effects [86,87,88]. It has been seen that the Mediterranean diet modulates the levels of certain miRNAs that could be involved in the regulation of the aforementioned biological processes. For example, high adherence to the Mediterranean diet has been associated with higher serum levels of miR-590 in adults with morbid obesity [64], a miRNA which has been related to anti-inflammatory effects, a reduction in lipid accumulation and the inhibition of atherosclerotic progression [89,90]. Although this was the only study which evaluated the impact of the Mediterranean diet as a whole, several studies have considered specific foods included in this dietary pattern, such as EVOO [56,57], nuts [51,52,53,54,55], grapes [67] and even plant-based diets [61,62,63]. Those studies showed that the intake of different plant-based foods can modulate the same miRNAs (Figure 2b). For instance, nut and EVOO consumption up-regulate circulating miR-192 [53,56], a miRNA associated with lipid and glucose metabolism [64,91,92], and down-regulate circulating miR-328 [53,56], which, according to the evidence, is related to cardiovascular disease [93,94]. miR-29, miR-106 and miR-181 are also examples of miRNAs modulated by several plant-based foods, and their biological effects are discussed below. Although the way miRNAs impact metabolic control is still not fully understood, their modulation may be one of the mechanisms through which the Mediterranean diet and plant-based foods improve health and reduce the risk of diseases.

### 4.3. Biological Effects of Diet-Modulated miRNAs

Many studies suggest the modulatory role of miRNAs in key physiological processes and their ensuing impact on health [95]. Particularly, they have been associated with glucose and lipid metabolism [79], which is fundamental to human homeostasis. Here, we have revised the potential of foods and diets to influence miRNA levels and, given the strong involvement of unbalanced diets in the development of metabolic diseases, further investigation into the physiological role of miRNAs could provide new molecular targets that contribute to their prevention.

**Figure 3:**
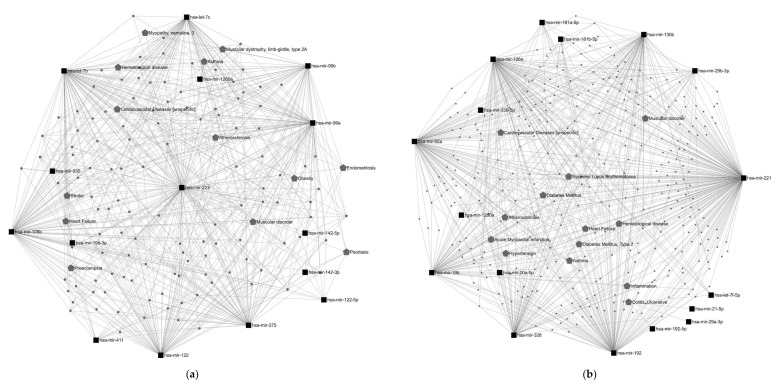
miRNA–disease interaction network. Network gene analysis was performed using miRNet 2.0 software [32]. Black squares represent the most relevant miRNAs modulated by (**a**) energy-controlled dietary patterns and (**b**) plant-based foods; gray pentagons represent diseases related to these miRNAs; bigger pentagons show diseases interacting with ≥3 miRNAs, excluding cancer-related diseases. In addition, a miRNA target network analysis was also performed using miRNet 2.0. A total of 11,928 target genes were found to be associated with miRNAs modulated by energy-controlled patterns, showing the miR-34 and let-7 families had the highest degree of interaction with them. Besides that, this set of miRNAs was significantly involved in 13 metabolic pathways, predominantly in angiogenesis (Figure 4a).

Studies on energy-controlled diets and plant-based foods have substantially shown their relevance in modulating endogenous miRNA levels. However, large differences were found regarding the miRNAs affected by different dietary patterns. This might be attributed to the metabolic effects of dietary nutrients on the body. Each nutrient present in the diet triggers distinct biological processes related to functions essential to our organism. The modulation of endogenous miRNA expression might contribute to the mechanisms underlying the specific functions of each nutrient. Consequently, each dietary pattern might be linked to a specific miRNA expression profile aimed at optimizing nutrient metabolism. Additionally, the evidence suggests that some bioactive compounds in food would be likely to directly change miRNA expression in a positive way, contributing to their health properties. For instance, curcumin, a flavonoid found in turmeric, has been attributed to anti-inflammatory and anti-tumorigenic properties, which are potentially mediated by miRNA activity, including miR-17, miR-20a and miR-27 [96]. The anti-inflammatory properties of resveratrol have also been linked to miRNA regulation. A study assessing resveratrol supplementation in men with type 2 diabetes and hypertension has reported the modulation of a set of miRNAs involved in the inflammatory response [97]. The phenolic compounds from nuts and extra virgin oil are also examples of miRNA activity modulators [96]. Although these findings contribute to our understanding of the role of miRNAs in the health-related properties of diet, the evidence is not sufficient to clearly determine how each dietary pattern influences fundamental biological processes through miRNA modulation. Here, we thoroughly examined the potential physiological functions of these two sets of miRNAs: energy-controlled patterns (Figure 2a) and plant-based foods (Figure 2b). To explore them, first, a miRNA–disease interaction network analysis was performed using the miRNet 2.0 software [32]. Only those miRNAs showing significant results in more than one study were included. The resulting network diagrams are shown in Figure 3. Regarding miRNA–disease interactions, the miRNet software displayed 100 outcomes, of which 57 were related to carcinomas and neoplasms. As miRNAs have been widely studied in relation to cancer and there is strong evidence of this, a large number of results on cancer were expected. Among the 47 remaining outcomes, cardiovascular diseases showed the strongest association with miRNAs modulated by energy-controlled patterns. Obesity, atherosclerosis and stroke also presented a high number of associations (Figure 3a). This highlights the implication of miRNAs in the mechanisms involved in cardiovascular disease development and other risk factors for metabolic syndrome and the need to balance caloric intake to prevent these diseases.

**Figure 4:**
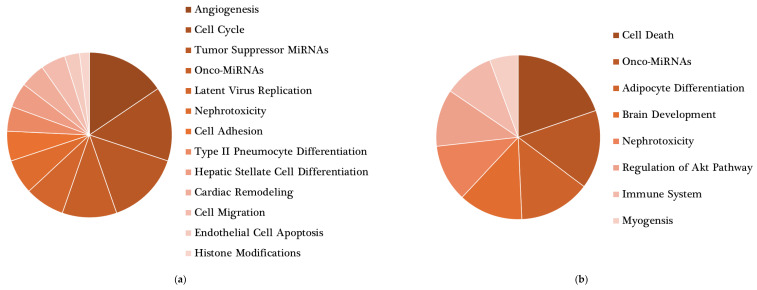
Main roles of target genes found in the network analysis. miRNet 2.0 software was used to determine relevant functions of miRNAs modulated by (**a**) energy-controlled dietary patterns and (**b**) plant-based foods. Only statistically significant (*p* < 0.05) outcomes are shown.

Concerning the set of miRNAs modulated by plant-based foods, the miRNet software also displays 100 diseases interacting with this set of miRNAs, half of them carcinomas and neoplasms. Among the non-cancer outcomes, diabetes, cardiovascular diseases, atherosclerosis and hypertension were some of the most connected in the network (Figure 3b). A total of 9,113 target genes were found. miR-20, miR-15, let-7 and miR-181 were the miRNAs with the strongest interaction. Eight metabolic pathways were significantly influenced by the miRNAs modulated by plant-based foods; among them, adipocyte differentiation and regulation of the AKT pathway appeared to be interesting in the context of diet and health (Figure 4b).

Of note, a few studies have evaluated the intake of animal-based foods, enabling a comparison with plant-based foods in terms of miRNA modulation. A study assessing the intake of red meat has found an association with miR-19 and miR-21, two miRNAs also modulated by nut [53] and EVOO [56,57] intake. Additionally, one article comparing the effects of plant-based and omnivorous diets reports a trend in miRNA expression, gradually changing its levels from vegan and vegetarian to omnivorous patterns [62]. This suggests that animal and plant origin food may modulate a similar set of miRNAs but in a different way. However, as certain studies evaluating similar dietary patterns obtain opposite results, a clear interpretation cannot be secured.

The network analysis pointed out the involvement of miRNA regulation in metabolic health. Particularly, the two sets of miRNAs analyzed were highly related to cardiovascular health, inflammation and the immune system. Conducting further studies on diet–miRNA and miRNA–health interactions may contribute to the development of nutritional strategies focused on preventing diseases through miRNA modulation.

### 4.4. Exogenous miRNAs from Dietary Sources

Apart from the regulatory role of diet in miRNA expression, it is also important to consider those miRNAs that can be directly ingested through dietary sources. They are found both in animals and plants, and in both kingdoms, miRNAs can regulate gene expression [98]. As plant miRNAs have been found in mammalian specimens, the evidence suggests that they can regulate cross-kingdom gene expression through dietary intake [98,99,100]. Mature plant-based miRNAs are present not only in raw vegetables but also in their cooked form (as in rice, wheat or potato) and can survive the gastrointestinal tract, even the acidic environment of the stomach [12]. Once exogenous miRNAs enter the intestinal epithelial stem cells, they can be packaged into vesicles and transported through the bloodstream, potentially affecting endogenous processes [101]. Although there is increasing evidence of exogenous miRNAs being acquired through dietary sources, their role as regulators of gene expression is still controversial [102]. Some authors consider that the presence of plant miRNAs in human samples is due to the contamination and oversensitivity of sequencing methods and also that the number of exogenous miRNAs is not enough to affect human gene expression [103,104].

The number of articles was insufficient to include the intake of exogenous miRNAs in the review. Nevertheless, some studies display their absorbability and their potential metabolic role; thus, we should not discard them. Researchers have detected significant levels of ath-miR-156a, osa-miR-168a and ath-miR-166a, three exogenous plant-derived miRNAs, in human serum [12]. Furthermore, in vitro analysis has shown that osa-miR-168a, which is abundant in rice and cruciferous vegetables, has a regulatory role in host gene expression, specifically as a modulator of liver-specific low-density lipoprotein receptor adapter protein 1 (LDLRAP1), which has a role in the removal of LDL from the plasma [12]. Another in vitro model shows that miR-156a, found in rice and green vegetables, can modulate the junction adhesion molecule-A (JAM-A), which is related to the inflammatory recruitment of mononuclear cells in the endothelium of atherosclerotic arteries [14]. Moreover, an in silico analysis which has examined four human datasets has identified 35 exogenous miRNAs in human milk exosomes belonging to 25 plant miRNA families [13].

Exogenous miRNAs acquired through the intake of species in the same kingdom are also noteworthy. Milk is a case in point. This fluid constitutes a relevant source of miRNAs for lactating progeny since they are transported in exosomes or vesicles and protected from degradation and digestion [105]. Humans can absorb biologically effective doses of miRNAs from mammals, as observed with cow milk [8,106]. Considerable amounts of two milk-based miRNAs (miR-29b and miR-200c) have been detected in the postprandial plasma of healthy adults after the consumption of bovine milk [106]. The absorbability of miRNAs from maternal milk has also been proposed [107,108,109] but is still under debate. In this context, the miRNA supply during breastfeeding is of great interest due to the importance of early nutrition to the metabolic programming of babies. Nutrition during the early stages of life has long-term consequences for health, and the metabolic adaptations induced in this critical period of child development can modulate susceptibility to metabolic disorders in adulthood [110,111,112]. Breastfeeding plays an important role in metabolic programming, and the function of miRNAs in metabolic programming should be considered. The evidence in humans is scarce, but animal models have demonstrated its involvement [113,114]. miR-148 is the most abundant miRNA in milk [109,115,116] and has been related to immune regulation, metabolism and development [109,117,118]. Among the articles included in this review, none of them have analyzed the miR-148 levels in milk, but in one of them, the intake of a single hypercaloric high-saturated-fat meal has been related to the PBMC levels of miR-148, that is, the underexpression of miR-148a and the overexpression of miR-148b [48]. Animal models also demonstrate the ability of diet to modulate miRNAs in milk. Higher levels of miR-222 and lower levels of miR-200a and miR-26a have been observed in milk from rats fed with a cafeteria diet (fat-rich hypercaloric diet) compared to controls [28].

In view of the above, further studies are needed to strengthen these data, especially to support the evidence of miRNAs being significantly absorbed from breast milk due to the importance of metabolic programming in early life stages.

## 5. Conclusions

This review underlines the ability of changes in dietary habits to regulate endogenous miRNA levels. Particularly, energy and fat content appeared to be key factors since they are the nutritional elements with more evidence supporting their role in miRNA modulation. However, the current studies are heterogeneous, which hinders the interpretation of the results jointly. The involvement of miRNAs in the regulation of biological processes and its potential impact on health have also been exposed. Animal models highlight their metabolic repercussions [29,30,119], but the evidence in humans is scarce. In this context, two sets of miRNAs emerged as linked to energy-controlled diets and plant-based foods, and the network analysis revealed its role in cardiometabolic health. Further studies are needed to clarify the consequences of diet on human miRNAs and to reliably identify their relationship with health conditions. Notwithstanding, instead of considering specific miRNAs separately, it would be interesting to identify miRNA profiles related to particular metabolic pathways and determine the dietary patterns that could balance them.

## Figures and Tables

**Table 1:** Eligibility criteria of the systematic review.

Inclusion Criteria	Exclusion Criteria
Studies conducted in humans	Studies not conducted in humans
Observational or interventional studies	Reviews and other design types
Studies evaluating dietary modulation of miRNA expression	Studies evaluating supplementation rather than food intake
Studies performed in adults	Population age under 18
Studies on healthy population with or without obesity	Study population with any other disease

**Table 2:** General characteristics of included studies.

Author and Publication Date	Population (F/M)	Health Status	Study Design	Dietary Strategy	Method of Analysis	Sample Analyzed	Outcomes
Giardina, 2019 [33]	103 adults (82/21), 30–60 years	With overweight/obesity (27–35 kg/m^2^)	Randomized, parallel-group, controlled trial	6-month study, three groups of energy-restricted diets (500 kcal/day restriction): moderate-carbohydrate and low-glycemic-index diet (LGI), moderate-carbohydrate and high-glycemic-index diet (HGI) and low-fat and high-glycemic-index diet (LF)	Array screening and validation using qPCR	Plasma	LGI vs. HGI: ↓ hsa-miR-361 After LGI: ↓ hsa-miR-139-3p ↓ hsa-miR-411 ↓ hsa-miR-432 ↓ hsa-miR-99b ↓ hsa-miR-340 ↓ hsa-miR-423-5p ↓ hsa-miR-361 ↓ hsa-let-7c After HGI: ↓ hsa-miR-139-3p ↓ hsa-miR-340 After LF: ↓ hsa-miR-139-3p ↓ hsa-miR-432 ↓ hsa-miR-423-5p
Margolis, 2017 [34]	16 men, 60–75 years	With overweight (25–35 kg/m^2^)	Randomized, parallel-group, triple-blinded trial	35-day study: 7-day weight maintenance and 28-day 30% energy restriction periods	qPCR	Serum	Energy restriction: ↑ miR-133a-3p ↑ miR-133b
Assmann, 2020 [35]	103 adults	Intervention groups with obesity (30–40 kg/m^2^) and control group with normal weight	Randomized, parallel-group trial	16-week weight loss intervention, three groups with 30% energy restriction: moderately high-protein diet (n = 38, 40% carbohydrate, 30% protein, 30% fat), low-fat diet (n = 40, 60% carbohydrate, 18% protein, 22% fat) and control (n = 25)	qPCR array	Plasma	Responders vs. non-responders to low-fat diet: ↓ hsa-miR-130a-3p ↓ hsa-miR-142-5p ↓ hsa-miR-144-5p ↓ hsa-miR-15a-5p ↓ hsa-miR-221-3p ↓ hsa-miR-29c-3p ↑ hsa-miR-22-3p
Duggan, 2022 [36]	192 postmenopausal women, 50–75 years	With overweight/obesity	Randomized, parallel-group, single-blinded, controlled trial	12-month weight loss intervention in four groups: restricted diet, exercise, diet + exercise and control (no intervention)	NanoString nCounter technology	Plasma	Weight loss intervention vs. control: ↓ miR-122
Heianza, 2022 [37]	495 adults	Healthy and with overweight	Randomized trial	2-year study, four groups of weight loss diets: low-fat average-protein diet, low-fat high-protein diet, high-fat average-protein diet and high-fat high-protein diet	RNA-seq	Plasma	High-fat and high-protein vs. low-fat and average-protein: ↓ hsa-miR-128-1-5p
Hess, 2020 [38]	85 adults (55/30), 18–60 years	With overweight/obesity (28–45 kg/m^2^)	Randomized, parallel-group, double-blinded trial	12-week study, two weight loss intervention groups, both with an energy deficit of 500 kcal/day: fiber supplementation (20 g/day) and control (no supplement)	qPCR array	Serum	After both diets: ↓ hsa-miR-122-5p ↓ hsa-miR-193a-5p ↑ hsa-miR-126a-3p ↑ hsa-miR-222-3p
Jayasooriya, 2022 [39]	27 adults, 18–60 years	With overweight (25–34.9 kg/m^2^)	Interventional single-arm pilot study	6-week weight loss intervention: diet (250 kcal/d energy restriction) and exercise	miRNA sensor *iLluminate*	Serum	Post vs. pre: ↓ hsa-let-7b ↓ hsa-miR-99a
Kristensen, 2017 [40]	19 adults (10/9)	With morbid obesity (≥40 kg/m^2^)	Interventional study	15-week weight loss intervention: exercise and hypocaloric diet	Array screening and validation using qPCR	Subcutaneous adipose tissue	↑ hsa-miR-29a-3p ↑ hsa-miR-29a-5p ↓ hsa-miR-20b-5p
Milagro, 2013 [41]	10 women	With obesity (35.6 kg/m^2^)	Interventional study	8-week weight loss intervention (800–880 kcal/day). Two groups: responders (weight loss > 5%) and non-responders (weight loss < 5%)	RNA-seq screening and validation using qPCR	PBMC	Non-responders: ↑ hsa-miR-935 ↑ hsa-miR-4772-3p ↓ hsa-miR-223 ↓ hsa-miR-224 ↓ hsa-miR-376b
Müller, 2020 [42]	46 adults (26/20), 18–72 years	With obesity (>30 kg/m^2^)	Interventional study	3-month weight loss intervention: 800 kcal/day (only protein shake)	RNA-seq screening and validation using qPCR	Plasma	↓ hsa-miR-25-3p ↓ hsa-miR-93-5p ↓ hsa-miR-106b-3p
Parr, 2016 [43]	40 adults (26/14), 35–59 years	With obesity (27–40 kg/m^2^)	Randomized, parallel-group, controlled trial	16-week weight loss intervention: 250 kcal/day energy restriction and exercise. Two groups: HiRes (>10% body mass loss, n = 22) and LoRes (<5% body mass loss, n = 18)	qPCR array	Plasma	LoRes vs. HiRes: ↑ hsa-miR-935 Both groups: ↑ hsa-miR-221-3p ↑ hsa-miR-223-3p
Tabet, 2016 [44]	47 men, 20–65 years	With obesity (32 kg/m^2^)	Randomized, parallel-group, controlled trial	12-week weight loss intervention. Two groups of hypocaloric diets: high-protein diet (30% of energy, n = 20) and normal-protein diet (20% of energy, n = 27)	qPCR	HDL-fraction of serum samples	High-protein diet: ↓ miR-223
Lilja, 2021 [45]	54 adults (35/16), 23–75 years	Healthy	Interventional study	5-day study, two groups: fasting (5 days of only liquids) and control (non-fasting)	qPCR	Stool	Fasting: ↑ let-7b-5p ↓ miR-34a-5p
Ravanidis, 2021 [46]	32 adults (10/22), 18–70 years	With overweight/obesity (28 kg/m^2^)	Interventional single-arm study	10-day fasting period (250 kcal/day)	qPCR	Plasma	↓ hsa-miR-19b-3p ↓ hsa-miR-22-3p ↓ hsa-miR-142-3p ↓ hsa-miR-143-3p ↓ hsa-miR-145-5p ↑ hsa-miR-122-5p ↑ hsa-miR-126-3p
Saini, 2022 [47]	9 older adults (6/3), ≥65 years	With overweight	Interventional single-arm study	4 weeks with 16 h per day of fasting	RNA-seq	Serum	After intervention: ↑ miR-623 ↑ miR-4303 ↑ miR-7162-3p ↑ miR-411-5p ↑ miR-5682 ↑ miR-4513 ↓ miR-4649-5p ↓ miR-2467-3p ↓ miR-543 ↓ miR-301a-3p ↓ miR-3132 ↓ miR-19a-5p ↓ miR-495-3p ↓ miR-4761-3p
Lopez, 2018 [48]	9 men, 18–23 years	Healthy	Randomized, crossover, double-blinded trial	A single high-saturated-fat meal (800 kcal, 77% fat, 23% carbohydrate)	Array screening and validation using qPCR	PBMC	Postprandial: ↓ hsa-miR-613 ↓ hsa-miR-629-3p ↓ hsa-miR-24-2-5p ↓ hsa-miR-555 ↓ hsa-miR-148a-5p ↓ hsa-miR-621 ↓ hsa-miR-875-3p ↓ hsa-miR-513c-5p ↓ hsa-miR-1226 ↑ hsa-miR-653 ↑ hsa-miR-19b-1-5p ↑ hsa-miR-363-5p ↑ hsa-miR-885-3p ↑ hsa-miR-339-3p ↑ hsa-miR-938 ↑ hsa-miR-148b-5p ↑ hsa-miR-593-5p ↑ hsa-miR-200b-5p
Quintanilha, 2022 [49]	12 adults (7/5), 25–45 years	Healthy	Randomized, crossover trial	A single high-fat high-carbohydrate meal + water/orange juice/isocaloric beverage with 1-week washouts	qPCR	Plasma	Meal + orange juice vs. water: ↑ hsa-miR-375 Meal + glucose vs. water: ↓ hsa-miR-205-3p
Quintanilha, 2020 [50]	11 women, 20–40 years	Healthy	Interventional trial	A single high-fat high-saturated meal (1067 kcal)	qPCR array	Plasma	Postprandial: ↑ hsa-miR-200c-3p ↑ hsa-miR-143-5p ↑ hsa-miR-200b-3p ↑ hsa-miR-143-3p ↑ hsa-miR-375 ↑ hsa-miR-145-5p ↓ hsa-miR-1260a ↓ hsa-miR-92b-3p ↓ hsa-miR-205-5p
Gil-Zamorano, 2022 [51]	8 adults, 63–79 years	Healthy	Randomized, parallel-group, single-blinded, controlled trial	1-year study, two groups: walnut supplementation (30–60 g/day) and control (abstaining from walnuts)	Array screening and validation using qPCR	Serum	↑ hsa-miR-551a
López de las Hazas, 2021 [52]	211 subjects, 63–79 years	Healthy	Randomized, parallel-group, single-blinded, controlled trial	1-year study, two groups: walnut supplementation (n = 101, 30–60 g/day) and control (n = 110, abstaining from walnuts)	Screening in 40 pools of samples and validation using qPCR	Plasma	Walnuts: ↑ hsa-miR-32-5p ↑ hsa-miR-29b-3p
Ortega, 2015 [53]	30 adults (22/8), 30–50 years	With obesity (30–35 kg/m^2^)	Interventional study	8-week study: normocaloric diet enriched with PUFA (30 g/day almonds and walnuts)	Array screening and validation using qPCR	Plasma	↓ miR-328 ↓ miR-330-3p ↓ miR-221 ↓ miR-125a-5p ↑ miR-192 ↑ miR-486-5p ↑ miR-19b ↑ miR-106a ↑ miR-130b ↑ miR-18a ↑ miR-769-5p
Reis, 2019 [54]	54 women, 18–55 years	With overweight/obesity (≥27.5 kg/m^2^)	Randomized, parallel-group, controlled trial	2-month study. Two groups: Brazil nut (1 Brazil nut/day, n = 29) and control (no Brazil nuts, n = 25)	qPCR	Plasma	Brazil nut intake: ↑ miR-454-3p ↑ miR-584-5p
Salas-Huetos, 2018 [55]	98 men, 18–35 years	Healthy	Randomized, parallel-group, single-blinded, controlled trial	14-week study, two groups: nuts (Western diet + 60 g/day nuts) and control (Western diet avoiding nuts)	Array for screening and validation	Sperm	Nuts: ↓ hsa-miR-34b-3p
Daimiel, 2020 [56]	12 adults (6/6), 22–60 years	Healthy	Randomized, crossover, double-blinded, controlled trial	30 mL of 3 polyphenol-enriched EVOOs after 12 h of fasting: low-EVOO (250 mg/kg of oil), medium-EVOO (500 mg/kg) and high-EVOO (750 mg/kg) diets	qPCR array	Plasma	EVOO: ↓ l hsa-let-7e-5p ↓ hsa-miR-328a-3p ↓ hsa-miR-10a-5p ↓ hsa-miR-21-5p ↓ hsa-miR-26b-5p ↑ hsa-miR-17-5p ↑ hsa-miR-20a-5p ↑ hsa-miR-192-5p
D’Amore, 2016 [57]	24 adults (12/12)	12 healthy and 12 with metabolic syndrome	Interventional study	Two interventions: single intake of high-polyphenol EVOO and low-polyphenol EVOO (50 mL) after 12 h fasting and a 1-week washout with no olive oil intake	Array screening and validation using qPCR	PBMC	High-polyphenol EVOO in healthy adults: ↑ miR-23b-3p ↑ miR-519b-39 ↓ miR-146b-5p ↓ miR-19a-3p ↓ miR-181b-5p ↓ miR-107 ↓ miR-769-5p ↓ miR-192-5p
Cannataro, 2019 [58]	36 adults (18/18), 31–58 years	With obesity (>30 kg/m^2^)	Interventional study	6 weeks of a ketogenic diet	NanoString nCounter technology	Serum	Ketogenic diet: ↑ hsa-let-7b-5p ↑ hsa-miR-143-3p ↓ hsa-miR-504-5p
Desgagné, 2016 [59]	9 men, 20–59 years	Healthy	Randomized, crossover, double-blinded, controlled trial	Three 4-week interventions with 3-week washout periods: high-iTFA (10.2 g industrial TFA/2500 kcal, 3.7% energy), high-rTFA (10.2 g dairy and meat TFA/2500 kcal, 3.7% energy) and low-TFA (2.2 g/2500 kcal, 0.8% energy)	qPCR	HDL plasma-fraction	No significant results
Ferrero, 2021 [60]	120 adults (72/48)	Healthy	Observational study	Equal % of vegans, vegetarians and omnivores, diet > 1 year. Food frequency questionnaires	RNA-seq	Plasma	No significant results
Liu, 2020 [61]	96 adults (53/43), ≥60 years	Healthy	Observational study	31 non-vegetarians, 15 vegans, 32 lacto-vegetarians and 18 semi-vegetarians. Food frequency questionnaires	RNA-seq	Plasma	Vegetarians: ↑ hsa-miR-3661 ↑ hsa-miR-320c ↑ hsa-miR-29a-3p ↑ hsa-miR-320b ↑ hsa-miR-204-3p ↓ hsa-miR-132-5p
Tarallo, 2014 [62]	24 adults (15/9), 21–60 years	Healthy	Observational study	8 vegans, 8 vegetarians, 8 omnivorous	qPCR	Stool and plasma	Vegan/vegetarian: ↑ hsa-miR-92a
Tarallo, 2022 [63]	120 adults (72/48)	Healthy	Observational study	Vegan, vegetarian and omnivorous, diet >1 year. Food frequency questionnaires	RNA-seq	Plasma and stool	No significant results in plasma Vegan/vegetarians: ↓ hsa-miR-636 ↓ hsa-miR-4488-3p ↓ hsa-miR-4739
Fontalba-Romero, 2021 [64]	58 adults (41/17)	With morbid obesity (≥40 kg/m^2^)	Observational study	MEDAS questionnaire to determine the adherence to a Mediterranean diet	Array screening and validation using qPCR	Serum	High Med diet adherence: ↑ miR-590
Humphreys, 2014 [17]	23 adults (6/17), 50–75 years	Healthy	Randomized, crossover, controlled trial	Two 4-week interventions with 4-week entry and washouts: HRM (300 g/day meat) and HRM + HAMSB (300 g/day meat + 40 g/day butyrylated high amylose starch)	qPCR	Rectal mucosa	HRM: ↑ hsa-miR-19a-3p ↑ hsa-miR-19b-3p ↑ hsa-miR-21-5p HRM + HAMSB: ↓ hsa-miR-17-5p ↓ hsa-miR-19a-3p ↓ hsa-miR-19b-3p ↓ hsa-miR-20a-5p ↓ hsa-miR-92a
Ramzan, 2019 [65]	31 men, ≥70 years	Healthy	Randomized, parallel-group, single-blinded trial	10-week study, two groups: RDA (0.8 g protein/kg body weight/day) and 2RDA (1.6 g/kg body weight/day)	RNA-seq screening and validation using qPCR	Plasma	2RDA: ↓ hsa-miR-125b-5p ↓ hsa-miR-100-5p ↓ hsa-miR-99a-5p ↓ hsa-miR-23b-3p ↓ hsa-miR-203a
Shin, 2020 [66]	10 women, 50–60 years	With overweight 25–30 kg/m^2^)	Randomized, parallel-group trial	2-week study. Two groups: k-diet (traditional Korean diet) and control (Westernized Korean diet)	Array screening and validation using qPCR	Plasma and saliva	K-diet, plasma: ↓ hsa-miR-26a-5p ↓ hsa-miR-126-3p Control, plasma: ↓ hsa-miR-25-3p K-diet, saliva: ↓ hsa-miR-92-3p ↓ hsa-miR-122a-5p Control, saliva: ↓ hsa-miR-31-5p
Tutino, 2021 [67]	40 adults (29/11), 30–65 years	With overweight (25–30 kg/m^2^)	Randomized, parallel-group, single-blinded, controlled trial	21-day study. Two groups: grape group (5 g/day fresh grape/kg body weight) and control (abstaining from grapes)	qPCR array	Serum	Grape group: ↑ hsa-miR-208a-3p ↑ hsa-miR-33a-5p ↓ hsa-miR-181a-5p ↓ hsa-miR-30e-5p ↓ hsa-miR-30d-5p ↓ hsa-miR-335-5p ↓ hsa-miR-222-3p ↓ hsa-miR-15a-5p ↓ hsa-miR-421 ↓ hsa-miR-339-5p ↓ hsa-miR-378a-3p ↓ hsa-let-7f-5p ↓ hsa-miR-29b-3p ↓ hsa-miR-106b-3p ↓ hsa-miR-324-5p ↓ hsa-miR-1260a ↓ hsa-miR-365a-3p ↓ hsa-miR-155-5p ↓ hsa-miR-335-3p ↓ hsa-miR-200c-3p
Capetini, 2023 [68]	20 women, 18–40 years	With overweight (25–29.9 kg/m^2^)	Interventional study	4 weeks consuming 500 mL/d of blood orange juice	Array screening and validation using qPCR	Plasma and PBMC	Plasma: ↑ hsa-miR-144-3p PBMC: ↑ hsa-miR-144-3p ↑ hsa-miR-424-5p ↑ hsa-miR-130b-3p ↓ hsa-let-7f-5p ↓ hsa-miR-126-3p

Legend: F/M, female/male; PBMC, peripheral blood mononuclear cell; HDL, high-density lipoprotein; PUFA, polyunsaturated fatty acids; EVOO, extra virgin olive oil; TFA, trans fatty acids; MEDAS, Mediterranean Diet Adherence Score; HRM, high red meat; HAMSB, supplementation with butyrylated resistant starch; RDA, recommended dietary allowance; ↓, decreased level; ↑, increased level.

**Table 3:** Overview of human endogenous miRNAs modulated by diet in ≥2 studies indicating the source used to measure miRNA expression. The most relevant dietary patterns are included. miRNAs are grouped by family.

miRNA	Energy Restriction	Fasting	High-Fat Meal	Nuts	EVOO	Vegetarian Diet	Grape	Orange Juice	Red Meat
let-7	↓ plasma [33] ↓ serum [39]	↑ stool [45]			↓ plasma [56]		↓ serum [67]	↓ PBMC [68]	
miR-19		↓ plasma [46] ↓ serum [47]	↑ PBMC [48]	↑ plasma [53]	↓ PBMC [57]				↑ rectal mucosa [17]
miR-29	↓ plasma [35] ↑ adipose tissue [40]			↑ plasma [52]		↑ plasma [61]	↓ serum [67]		
miR-92			↓ plasma [50]			↑ plasma [62]			
miR-122	↓ serum [38] ↓ plasma [36]	↑ plasma [46]							
miR-126	↑ serum [38]	↑ plasma [46]						↓ PBMC [68]	
miR-20	↓ adipose tissue [40]				↑ plasma [56]				
miR-99	↓ plasma [33] ↓ serum [39]								
miR-106	↓ plasma [42]			↑ plasma [53]			↓ serum [67]		
miR-130	↓ plasma [35]			↑ plasma [53]				↑ PBMC [68]	
miR-143		↓ plasma [46]	↑ plasma [50]						
miR-192				↑ plasma [53]	↑ plasma [56] ↓ PBMC [57]				
miR-200			↑ PBMC [48] ↑ plasma [50]				↓ serum [67]		
miR-221	↓ plasma [35] ↑ plasma [43]			↓ plasma [53]					
miR-223	↓ PBMC [41] ↓ serum HDL [44] ↑ plasma [43]								
miR-15	↓ plasma [35]						↓ serum [67]		
miR-21					↓ plasma [56]				↑ rectal mucosa [17]
miR-22	↑ plasma [35]	↓ plasma [46]							
miR-34		↓ stool [45]		↓ sperm [55]					
miR-142	↓ plasma [35]	↓ plasma [46]							
miR-144	↓ plasma [35]							↑ PBMC [68]	
miR-145		↓ plasma [46]	↑ plasma [50]						
miR-181					↓ PBMC [57]		↓ serum [67]		
miR-205			↓ plasma [50] ↓ plasma [49]						
miR-222	↑ serum [38]						↓ serum [67]		
miR-328				↓ plasma [53]	↓ plasma [56]				
miR-339			↑ PBMC [48]				↓ serum [67]		
miR-375			↑ plasma [50] ↑ plasma [49]						
miR-411	↓ plasma [33]	↑ serum [47]							
miR-769				↑ plasma [53]	↓ PBMC [57]				
miR-935	↑ PBMC [41] ↑ plasma [43]								
miR-1260			↓ plasma [50]				↓ serum [67]		

Legend: EVOO, extra virgin olive oil; ↓, down-regulation; ↑, up-regulation; PBMC, peripheral blood mononuclear cell; HDL, high-density lipoprotein.
